# Editorial: Human-centered robot vision and artificial perception

**DOI:** 10.3389/frobt.2024.1406280

**Published:** 2024-07-08

**Authors:** Qing Gao, Xin Zhang, Chunwei Tian, Hongwei Gao, Zhaojie Ju

**Affiliations:** ^1^ School of Electronics and Communication Engineering, Sun Yat-sen University, Guangzhou, China; ^2^ Shenyang Institute of Automation, Chinese Academy of Sciences, Shenyang, China; ^3^ School of Computing, University of Portsmouth, Portsmouth, United Kingdom; ^4^ School of Software, Northwestern Polytechnical University, Xi’an, China; ^5^ Institute of International Engineering, Shenyang Ligong University, Shenyang, China

**Keywords:** robot vision, AI, human-robot interaction, deep learning, big data

**FIGURE 1:**
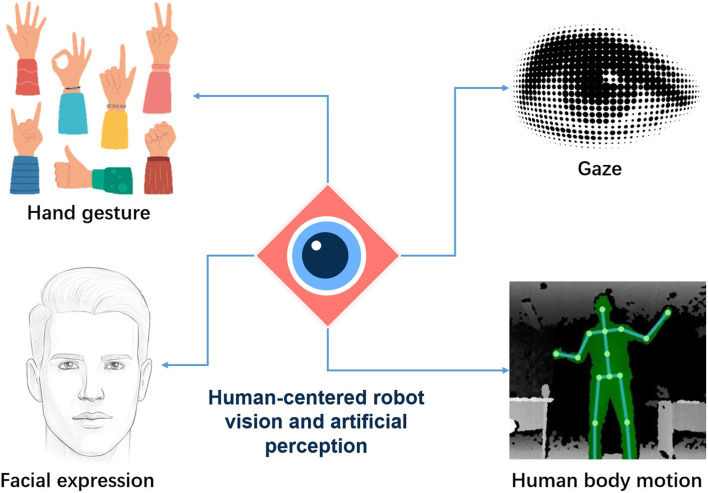
Human-centered robot vision and artificial perception.

In the daily life of human beings, more than 80% of their awareness of the surrounding environment relies on visual information, and the eyes are the most important sensory system of the human body. Meanwhile, human intentions and states are mostly reflected in non-verbal communication or body language behaviors. Intelligent perception of human modalities can recognize human states and intentions and provide reasonable feedback in virtual reality, driver monitoring systems, patient visual diagnosis, and human-computer interaction applications. As shown in Figure 1, human-centered robot vision and artificial perception mainly includes: gesture visual perception and interaction, body movement visual perception and interaction, gaze direction visual perception and interaction, facial expression visual perception and interaction, and human-object interaction visual perception. Driven by deep learning and big data, artificial perception has made great progress in both theory and application. We publish this Research Topic to bring together the latest theoretical findings and experimental results in this field. Of all the submissions in this Research Topic, four manuscripts were accepted after a standard review process. Below we present a brief review of the published articles.

Koller et al. investigated robotic gaze aversion and its effects on human behavior and attitudes. When humans are asked difficult questions, adults and children tend to avert their eyes at certain points in the interaction. Human-inspired gaze parameters have been used to implement gaze behavior for humanoid robots in conversational settings and to improve user experience (UX). However, it is unclear how deviations from human-inspired gaze parameters affect UX. Based on eye-tracking, interaction duration, and self-reported attitude measures, the authors investigated the impact of non-human-inspired gaze duration on participants’ UX in a conversational setting. The authors demonstrated that the results of systematically varying the gaze aversion rate (GAR) of a humanoid robot over a wide range of parameters (from 0.1 to 0.9). They found that a low GAR leads to shorter interaction durations and that human participants change their GAR to mimic the robot. Moreover, at the lowest gaze aversion setting, participants did not stare back as expected, suggesting that a high mutual gaze is not always a sign of high comfort. These research findings are useful for designing a robot gaze behavior for human-robot interaction. Wang and Chahl investigated the image-based heart rate estimation method, namely, imaging-photoplethysmography (iPPG), using three-dimensional (3D) human simulation. Due to the lack of high-quality human datasets, the authors propose an enhanced 3D human model with dynamically similar cardiac signals to those of a real person, which can be used to create a simulated dataset for cardiac signal extraction. The authors integrated all subject variables (e.g., facial expression, blinking, skin type, body activity, etc.) and environmental variables (light changes) into the simulated environment, and body motion caused by involuntary movements and breathing into the 3D human model to make the 3D model closer to the real scene. The authors evaluated the performance of five well-known traditional iPPG methods and four deep learning iPPG methods on a set of 3D human models with different appearances against real database videos. They found that the time and frequency domain signals from the 3D human body were in good agreement with the data from the comparison group (real human video) by various iPPG tests. Zhang et al. studied a vision-based trajectory teaching method by recognizing the finger trajectory. It consists of a color camera (RGB), a 3D sensor (D) and a thermal camera (T) for multimodal point cloud (RGB-D-T) perception. Since touching an object with a finger results in a slight temperature change of the object’s surface, the authors use the 3D thermal trajectory to represent the robot’s motion trajectory in the robot teaching mode. Here, the finger movement process can be considered as a heat transfer process caused by a moving Gaussian point heat source. The authors used RandLANet to perform hand/object semantic segmentation on multimodal 3D image data containing temperature, color and geometric features. Meanwhile, based on the heat transfer equation, the authors can analyze the residual heat on the object surface and predict the trajectory more accurately. Zhang and Czarnuch investigated the point cloud registration and completion from two or more sensors with arbitrary relative perspectives in a challenging indoor scenario with human motion. Since the human walks on the ground/floor, the authors located the ground planes in each set of sensors by finding all moving objects and aligning pairs of point clouds to the common ground plane. Based on this constraint, the authors reduced the complexity of the 3D point cloud completion from six unknowns (translation along the *x*-, *y*-, and *z*-axis and roll, pitch, and yaw rotations) to three unknowns (translation along the *x*- and *z*-axis and yaw rotation). Meanwhile, the authors extract all the humans from each frame and generate a three-dimensional human walking sequence in a time series using a histogram-based approach. Meanwhile, the center of mass point of each human body is calculated to improve the accuracy and performance. The Fréchet distance between two walking paths is used to match them and the 2D iterative closet point is used to find the reduced three unknowns of the transformation matrix for the final alignment. The authors tested their algorithm and achieved a success rate of 96.2% (25 of 26 trials) using a public dataset and their own dataset.

Finally, we hope that this Research Topic can provide inspiration for the research on human-centered robot vision and artificial perception

